# Treatment of acute hypernatremia caused by sodium overload in adults: A systematic review

**DOI:** 10.1097/MD.0000000000028945

**Published:** 2022-02-25

**Authors:** Takahiro Goshima, Teruhiko Terasawa, Mitsunaga Iwata, Asako Matsushima, Tomonori Hattori, Hiroshi Sasano

**Affiliations:** a Department of Emergency Medicine and General Internal Medicine, Fujita Health University School of Medicine, 1-98 Dengakugakubo, Kutsukakecho, Toyoake, Aichi, Japan; b Clinical Department of Emergency Medicine, Nagoya City University Hospital, 1 Kawasumi, Mizuhocho, Mizuhoku, Nagoya, Aichi, Japan.

**Keywords:** hypernatremia, osmolality, rapid correction, soy sauce ingestion, systematic review

## Abstract

**Background:**

Rapid-onset, acute hypernatremia caused by sodium overload is a rare, life-threatening condition. Although experts recommend rapid correction of sodium concentration [Na] based on pathophysiological theories, only a few reports have documented the specific details of sodium correction methods. The objective of this study was to systematically review the reported treatment regimens, achieved [Na] correction rates, and treatment outcomes.

**Methods:**

PubMed, Ichushi-database, and references without language restrictions, from inception to January 2021, were searched for studies that described ≥1 adult (aged ≥18 years) patients with rapid-onset hypernatremia caused by sodium overload, whose treatment was initiated ≤12 hours from the onset. The primary outcome of interest was the [Na] correction rate associated with mortality.

**Results:**

Eighteen case reports (18 patients; median [Na], 180.5 mEq/L) were included. The cause of sodium overload was self-ingestion in 8 patients and iatrogenic sodium gain in 10 patients; baseline [Na] and symptoms at presentation were comparable for both groups. Individualized rapid infusion of dextrose-based solutions was the most commonly adopted fluid therapy, whereas hemodialysis was also used for patients already treated with hemodialysis. The correction rates were more rapid in 13 successfully treated patients than in 5 fatal patients. The successfully treated patients typically achieved [Na] ≤160 within 8 hours, [Na] ≤150 within 24 hours, and [Na] ≤145 within 48 hours. Hyperglycemia was a commonly observed treatment-related adverse event.

**Conclusion:**

The limited empirical evidence derived from case reports appears to endorse the recommended, rapid, and aggressive sodium correction using dextrose-based hypotonic solutions.

## 1 Introduction

Hypertonic sodium gain is a rare but important cause of rapid onset (typically minutes–hours) of acute hypernatremia (ie, hyperacute hypernatremia). Typical etiologies include inadvertent medical interventions such as rapid infusion of a large volume of sodium bicarbonate and accidental sodium ingestion.^[1]^ Hyperacute hypernatremia is generally symptomatic and manifests as weakness, restlessness, nausea, vomiting, coma, or seizures, which can cause brain hemorrhage or osmotic demyelination, potentially causing death.^[2]^

Treatment of hypernatremia typically involves infusion of hypotonic solutions such as 5% dextrose in water (D5W), or in rare occasions, hemodialysis, to lower serum sodium concentration [Na]. In experimentally induced hypernatremia, electrolytes enter brain cells and reduce brain water content within a few hours during the rapid adaptation phase, whereas compensation (of this reduction in brain water) by intracellular organic osmolytes takes several days (slow adaptation phase).^[1,3,4]^ Based on this pathophysiological theory, it is widely recognized that extremely rapid correction of chronic hypernatremia is dangerous as such rehydration induces cerebral edema that can result in uncal herniation.^[5]^ In contrast, rapid correction of [Na] in acute hypernatremia is believed to be safe^[5]^ because adaptation changes in theory remain reversible during the rapid adaptation phase. Therefore, experts recommend rapid infusion of D5W to immediately restore normal [Na] within 24 hours.^[5,6]^

However, because of this condition's relative rarity and potentially high mortality, the detailed methodology and safety of such rapid correction of hypernatremia have only been rarely documented.^[7–10]^ Therefore, to systematically review the treatment regimens, achieved [Na] correction rates, and treatment outcomes, we conducted a systematic review of published reports that explicitly described the pertinent information.

## 2 Methods

This systematic review followed the Preferred Reporting Items for Systematic Reviews and Meta-Analyses 2020 statement.^[11]^ There was no registration or protocol for this systematic review.

### 2.1 Literature search

We searched the PubMed and Igaku-Chuo-Zasshi (Ichushi) databases on January 11, 2021, with no time or language restrictions using search terms, including “hypernatremia”. The references of included studies were used to identify additional citations. The exact search strategies are available in the Supplemental Digital Content Text.

### 2.2 Eligibility criteria and selection process

We included reports with any study design that described ≥1 adult (aged ≥18 years) patients with hyperacute (defined as ≤12 hours) hypernatremia caused by sodium overload, whose treatment was initiated ≤12 hours from the onset. Our operational definition of sodium overload was hypertonic sodium gain caused by inappropriate or accidental administration of hypertonic sodium solutions (eg, hypertonic saline or sodium bicarbonate solutions) or self-ingestion or accidental ingestion of salt or salt solutions (eg, soy sauce or seawater). We included only reports that explicitly presented numerical data, from which the correction rates of [Na] at ≥1 time point ≤12 hours from the start of treatment, irrespective of the treatment details, could be estimated, and clinical outcomes. We excluded reports that did not explicitly describe both the initial and follow-up (<24 hours) [Na] values and reports of nonacute hypernatremia secondary to other causes, including unreplaced water losses from any causes, including hyperosmolar hyperglycemic state, burns, or central diabetes insipidus. One investigator (T.G.) screened abstracts, and 2 independent investigators (T.G. and T.T.) performed full-text review for all screened-in study reports to determine eligibility. Discrepancies were resolved through consensus.

### 2.3 Data collection

One reviewer (T.G.) extracted data; another reviewer (T.T.) verified all the data. We extracted the first author and publication year, the number of study participants, and follow-up duration(s) as the study characteristics; age, gender, pretreatment baseline [Na] ([Na]_0_), comorbidities, cause of hypernatremia, ingested or administered sodium, rates of sodium ingestion/administration, time from ingestion to treatment, and symptoms as the patient characteristics; the administered fluids and their infusion rates, cotreatments, if any, and correction rates (mEq/L/h) estimated based on the reported follow-up [Na]s and their timings as the treatment characteristics; and clinical and neurological outcomes and other complications. We estimated the correction rates for all paired [Na]s measured at 2 sequential time points, as long as the difference between the 2 points was ≥0.5 hours. Discrepancies were resolved through consensus.

### 2.4 Quality of reporting

Because no recommended tools were established to evaluate risk of bias in case reports, we evaluated only the compliance with the consensus-based case reporting guidelines,^[12]^ as a measure of the quality of reporting. We evaluated the presence or absence of the description of the component subitems for 5 key reporting items, that is, patient information, clinical findings, diagnostic assessment, therapeutic intervention, and follow-up and outcomes. We then evaluated the overall quality of reporting based on the ratings for the 5 items. One reviewer (T.G.) evaluated the quality of reporting; another reviewer (T.T.) verified all the results. Discrepancies were resolved via consensus.

### 2.5 Data synthesis

Our primary outcome of interest was the [Na] correction rate associated with mortality. The secondary outcomes included intracranial hemorrhage and treatment-related adverse events. We descriptively analyzed the data by creating tables and graphs. For visualizing sequential changes in individual-level [Na]s, we constructed a time-series plot. We then calculated the cumulative probabilities of achieving “target” [Na]s using the Kaplan–Meier method to appropriately analyze time-to-event data. After examining the time-series plot, we posthoc determined 4 target [Na]s, 145, 150, 155, and 160 mEq/L, for the time-to-event analysis. As all analyses were exploratory based on small-sized data, we did not conduct statistical tests.

### 2.6 Sensitivity analysis

For assessing the stability of results, we posthoc repeated the time-to-event analysis by additionally including the excluded reports due to insufficient data (no explicit descriptions on the initial and/or follow-up [Na] values or no follow-up [Na] data within 24 hours of treatment initiation). For studies that reported on a follow-up [Na] as “normal” only, we operationally imputed 145 mEq/L, which is the recommended target concentration for treating patients with acute hypernatremia.^[1,6]^ The nearest minimum or maximum integer was imputed for a description specifying a range of [Na] (eg, 169 mEq/L for >168 mEq/L).

### 2.7 Ethics approval

Under our institutional policy, institutional review board approval was not required for systematic reviews.

## 3 Results

### 3.1 Study selection

**Figure 1:**
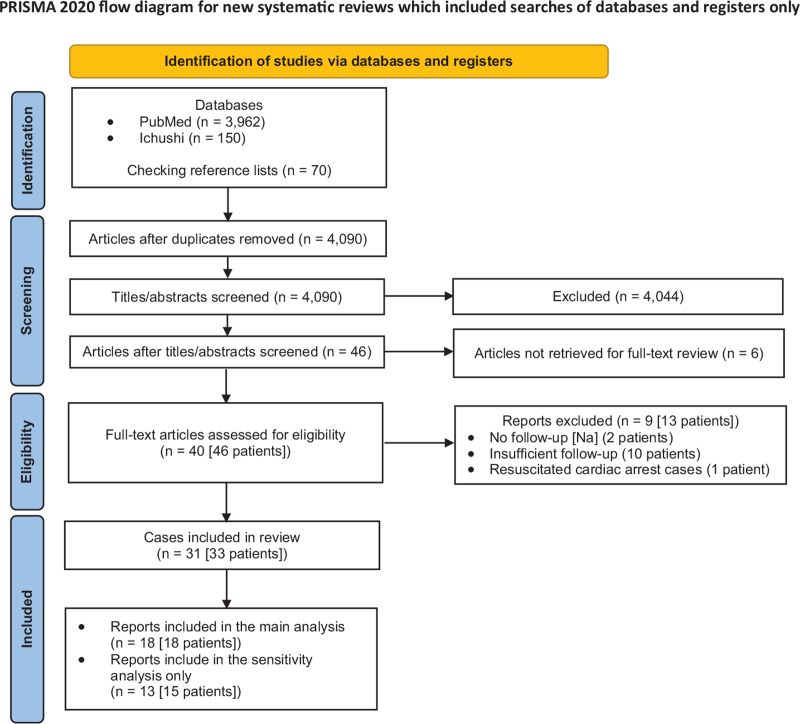
PRISMA flow diagram. [Na] = sodium concentration.

Figure 1 provides the details about the literature selection process. Upon a full-text review of 40 publications reporting 46 potentially eligible patients, we finally included 18 case reports reporting 18 patients^[7–10,13–26]^ in the main analysis. Additional 13 case reports with insufficient data reporting 15 patients were included only in the sensitivity analysis. Details of the 28 patients reported in 23 publications who were excluded from the main analysis are summarized in Table S1, Supplemental Digital Content.

### 3.2 Study and patient characteristics

Table 1 summarizes the study and patient characteristics. The publication year ranged from 1967 to 2020; 8 recent case reports (44%) were reported after 2010. The median age was 38 years (range, 19–73 years), and there were 13 female patients (72%). Rapid-onset hypernatremia was caused by self-ingestion of a large amount of sodium or sodium solutions in 8 (44%) patients, including 5 with a mental disorder, whereas iatrogenic sodium gain was the cause in the other 10 (56%) patients. The iatrogenic sodium gain included therapeutic irrigation or installation of hypertonic saline to focal lesions in 6 patients, intravenous infusion of a large amount of sodium in 2 patients, and dialysis errors in another 2 patients. Although a wide-ranging time duration between the onset of the hypernatremia cause and the start of treatment was reported (median 3.7 hours; range, <1–12 hours), the vast majority (15/18, 83%) received treatment at ≤6 hours. The 2 most common symptoms at presentation were seizures (8 patients, 44%) and coma (7 patients, 39%). These symptoms were similarly observed irrespective of the cause; 4 of 8 patients (50%) in the self-ingestion group and 4 of 9 patients (44%) in the iatrogenic group had seizures, and 4 of 8 patients (50%) in the self-ingestion group and 4 of 9 patients (44%) in the iatrogenic group were comatose. The median [Na]_0_ was 180.5 mEq/L (min–max, 167–209 mEq/L). The [Na]_0_ was >170 mEq/L in all patients, except for a female patient with a [Na]_0_ of 167 mEq/L in the iatrogenic group. Both groups had comparable severity of hypernatremia; the distribution of [Na]_0_ ranged from 172 to 209 mEq/L in the self-ingestion group and from 167 to 207.5 mEq/L in the iatrogenic group.

**Table 1:** Study and patient characteristics of included hyperacute hypernatremia cases.

Case no.	First author year [Ref.]	Age, yr (sex)	Pre-Tx [Na], mEq/L	Comorbidity	Cause of hypernatremia (total amount)	Time from onset to completion of the cause/time from onset to treatment, h	Acute symptoms (observed time point from hospital arrival, h)
1	Heckman 1967^[13]^	73 (F)	179	Hypercalcemia; musculoskeletal pain; constipation	IV sodium sulfate (total 726 mEq of sodium)	12/12	ND (0); striking neurologic improvement (48)
2	Roberts 1974^[14]^	26 (F)	172	Depression	Salt ingestion (150 g NaCl)^∗^ 1	<1/2	Drowsy (0)
3	Elisaf 1989^[15]^	24 (F)	178	Seizure	IV 15% NaCl (700 mL)	<1/<1	Coma (0)
4	Radonov 1989^[16]^	23 (F)	167	ND	Intrauterine administration of 25% NaCl for abortion (180 mL)	<1/<1	General cerebral symptoms without focal manifestations (0); clear consciousness (28)
5	Moder 1990^[7]^	41 (M)	209	Down syndrome; lymphoma; hepatitis B; seizure	Salt solution ingestion (∼90 g NaCl)	<1/4	Seizure (0)
6	Ellis 1997^[17]^	35 (M)	175	ND	Sea water ingestion (ND)^∗^	11/11	Delirium (0)
7	Albi 2002^[18]^	33 (F)	200	Hepatic hydatid cysts	Postsurgery liver cyst irrigations of 30% NaCl (∼3000 mL)	<8/<8	Seizure (0); confusion (0); agitation (0)
8	Ozcan 2003^[19]^	35 (F)	182	Hepatic hydatid cysts	Postsurgery liver cyst irrigations of 20% NaCl solution (ND)	<5/<5	GCS 6 (0); convulsion (ND)
9	Sakai 2004^[20]^	65 (F)	176	Schizophrenia	Soy sauce (17.5% NaCl) ingestion (1150 mL)	<1/4.5	Coma (0); seizure (0)
10	Odier 2010^[21]^	42 (M)	176	ND	“Dialysis error”	3.4/3.4	Seizure (0)
11	Carlberg 2013^[9]^	19 (M)	196	ND	Soy sauce (unclear concentration) ingestion (∼946 mL)	<1/2	Unresponsiveness (0); seizure (2); coma (2.5)
12	Bhosale 2015^[22]^	54 (M)	207.5	Metabolic acidosis; chronic renal failure	Inappropriate use of bicarbonate concentrate during dialysis	2/2	Seizure (0)
13	Conde 2015^[23]^	45 (F)	188	Depression; endometriosis; liver hydatid cysts	Postsurgery liver cyst irrigations of 20% NaCl (300 mL)	<3/<3	Nystagmus and fasciculations^†^ (0); seizure (16)
14	Izutani 2016^[8]^	36 (F)	192	Schizophrenia	Salt ingestion (200 g NaCl)	6/6	Senselessness (0); coma (1)
15	Anta 2017^[24]^	62 (F)	174	Hepatic hydatid cysts	Postsurgery liver cyst irrigations of 20% NaCl and hydroxide peroxide (50% mixed) (3500 mL)	3/3	Low level of consciousness (0); no neurological sequelae (6 d)
16	Zeng 2017^[25]^	28 (F)	188.8	Hepatic hydatid cysts	Postsurgery liver cyst irrigations of 30% NaCl (ND)	<1/1.7	Coma (0)
17	Miura 2019^[10]^	65 (F)	173	Depression	Soy sauce (15.3% NaCl) ingestion (600 mL)	3.5/5.75	Delirium (0), agitation (0)
18	Sakamoto 2020^[26]^	40 (F)	183	Schizophrenia	Soy sauce ingestion (70 g NaCl)	6–10/6–10	Seizure (0)

F = female, IV = intravenous, M = male, ND = no data.

∗ Sea water ingestion by near-drawing.

† Already on ventilator.

### 3.3 Treatment characteristics

Table 2 summarizes the adopted treatments. In total, 16 patients (88%) received various modes of intravenous fluid therapies. One patient also received electrolyte-free water through nasogastric tube in addition to intravenous fluid therapy (case 11). Of 11 (61%) patients treated with intravenous fluid administration alone, 13 had detailed data on a wide range of combination of the initially administered intravenous fluids and their infusion rates. The administered regimens were electrolyte-free water alone in 7 patients (D5W infused at 107–1000 mL/h [median, 400 mL/h] in 6 patients and 2.5% dextrose in water at 200 mL/h in 1 patient), hypotonic crystalloids (eg, a mixture of D5W and half saline solution) infused at 167–200 mL/hours in 2 patients, and isotonic crystalloids alone in 4 patients (normal saline infused at 250–1600 mL/h [median, 500 mL/h] in 3 patients and lactate Ringer solution at 200 mL/h in 1 patient). In the 4 patients who initially received an isotonic crystalloid fluid, the initial regimen was replaced with dextrose-based hypotonic solutions within 2 hours of treatment initiation. The subsequent infusion speed was modified based on the follow-up [Na]s; the maximum infusion rates during the first 48 hours ranged from 200 to 12,000 mL/h. Infusion rates were not reported in other 3 patients (one each for D5W, a hypotonic fluid, and normal saline). The other 3 patients (17%), 2 of whom had dialysis-related hypernatremia, were treated with hemodialysis. However, no report described the detailed dialysis methods.

**Table 2:** Therapeutic interventions and clinical outcomes of hyperacute hypernatremia.

Case no.	First author year [Ref.]	Age, yr (sex)	Pre-Tx [Na], mEq/L	Main fluid therapy (infusion rate, mL/h; timing from Tx, h) [other therapies]	Blood sample	Follow-up [Na], mEq/L (post-Tx time, h)	Correction rate, mEq/L/h (post-Tx time, h)	Final clinical outcomes (post-Tx d)	ICH (method, post-Tx d)	Major findings of the brain	Other findings
1	Heckman 1967^[13]^	73 (F)	179	D5W (107; 0–48)	Serum	177 (4) 175 (12) Normal (48)	−0.5 (0–4) −0.25 (4–12) ND	CR (ND)	ND	ND	Hypokalemia (ND)
2	Roberts 1974^[14]^	26 (F)	172	NS (250; 0–2); D5W (1333; 2–2.75); D5W (ND) [Mtl; FM; Dex; HD]	Plasma	162 (14) 152 (20)	−0.7 (0–14) −1.7 (14–20)	DTH (2)	+ (PME, 2)	Swollen and congested brain; patchy hemorrhage under the pia mater	
3	Elisaf 1989^[15]^	24 (F)	178	D5W (400–500; 0–6); D5W (200–250; 6–12)	Serum	157 (6) 143 (12) 138 (24)	−3.5 (0–6) −2.3 (6–12) −0.4 (12–24)	CR (30)	ND	ND	Hyperglycemia (max, 300 mg/dL); hypokalemia (min, 3.3 mEq/L)
4	Radonov 1989^[16]^	23 (F)	167	D5W (ND)	Plasma	158 (8) 148 (28)	−1.13 (0–8) −0.5 (8–28)	CR (25)	− (CT, ND)	Bilateral ischemic area	
5	Moder 1990^[7]^	41 (M)	209	D5W (1000; 0–1); D5W + ½NS (2000; 1–2); D5W + ½NS (600; 2–6); NS + HES (bolus)	Serum	201 (2) 191 (9) 182 (25) 168 (35) 155 (49) 145 (61)	−4.0 (0–2) −1.4 (2–9) −0.6 (9–25) −1.4 (25–35) −1.4 (35–49) −0.8 (49–61)	DTH (3)	+ (PME, 3)	Cerebral edema, hemorrhages in the midbrain and pons; tonsillar herniation	
6	Ellis 1997^[17]^	35 (M)	175	NS (500; bolus)^†^; D5W (ND)	Serum	174 (0.5) 164 (3.6) 159 (6.7) 154 (8.7) 147 (11.7) 142 (20) 142 (72)	−2 (0–0.5) −3.1 (0–3.6) −1.6 (3.6–6.7) −2.5 (6.7–8.7) −2.3 (8.7–11.7) −0.6 (11.7–20) ±0 (20–72)	CR (3)	ND	ND	
7	Albi 2002^[18]^	33 (F)	200	D2.5W (200; 0–24); D2.5W (100; 24–48)	Serum	187 (6) 179 (12) 166 (18) 154 (24)	−2.2 (0–6) −1.3 (6–12) −2.2 (12–18) −2 (18–24)	CR (7)	− (MRI, 42)	Normal	Hyperglycemia (221 mg/dL); body weight gain (+3 kg)
8	Ozcan 2003^[19]^	35 (F)	182	D5W (200; 0–6); D5W (200–250; 6–12)	Serum	170 (2) 156 (6) 156 (8) 153 (12) 153 (24) 142 (36) 148 (48)	−6.0 (0–2) −3.5 (2–6) ±0 (6–8) −0.8 (8–12) ±0 (12–24) −0.9 (24–36) +0.5 (36–48)	CR (2)	− (CT, 1)	ND	Hyperglycemia (max,492 mg/dL)
9	Sakai 2004^[20]^	65 (F)	176	HD (1–5); D5W (300; 0–5); NS (53; 5–24)	Plasma	146 (2) 140–146 (24–48)	−15 (0–2) ND	CR (7)	ND	ND	
10	Odier 2010^[21]^	42 (M)	176	HD (0–9.6)	ND	148 (9.6)	−2.9 (0–9.6);	CI (ND)	− (MRI, ND)	Extrapontine myelinolysis	
11	Carlberg 2013^[9]^	19 (M)	182	LRS (200; 0–∼1); D5W + ½NS (200; ∼1–2.5); D5W (12,000; 2.5–3); D5W (217; 3–27) [hydration via NG tube]	Plasma	187 (2) 196 (2.5) 170 (3) 145 (30)	+2.5 (0–2) +10 (2–2.5) −52 (2.5–3) −0.9 (3–30)	CR (9)	− (CT, 1); − (MRI, 3)	Dehydration Swollen brain with abnormal signal, and restricted diffusion of the right hippocampus	Hyperglycemia (max, 1116 mg/dL) and hypokalemia (min, 2.5 mEq/L)
12	Bhosale 2015^[22]^	54 (M)	207.5	HD (ND)	Serum	156 (8) 138 (24)	−6.4 (0–8) −1.1 (8–24);	CR (∼90)	− (MRI, 3)	Normal	
13	Conde 2015^[23]^	45 (F)	188	HoTS (200; 0–24) [FM] HoTS (217; 0–24); HoTS (100; 24–72])	Serum	177 (4) 153 (14) 155 (36) 144 (40) 146 (144)	−2.75 (0–4) −2.4 (4–14) +0.1 (14–36) −2.75 (36–40) +0.0 (40–144)	CR (∼60)	− (MRI, ND)	Central pontine myelinolysis	Hyperglycemia (max, 300 mg/dL) and hypokalemia (min, 3.3 mEq/L)
14	Izutani 2016^[8]^	36 (F)	192	ARS + ½NS (ND) [Mtl]	Serum	184 (2) 170 (10)^∗^ 185 (16) 196 (32) >180 (48)	−4.0 (0–2) −1.8 (2–10) +2.5 (10–16) +0.7 (16–32) NE	DTH (37)^∗^	+ (CT, 0.5); + (CT, 1.4)	Subarachnoid hemorrhage; Brain edema and herniation	
15	Anta 2017^[24]^	62 (F)	174	D5W and ½NS (167; 0–24)	Serum	179 (1) 164 (24) 148 (72)	+5 (0–1) −0.65 (1–24) −0.33 (24–72)	CR (6)	− (CT, 3)	Normal	Hyperglycemia (max, 173 mg/dL)
16	Zeng 2017^[25]^	28 (F)	188.8	NS (ND), NS + D5W + LRS (ND) [Mtl; FM]	ND	183 (<1) 169.9 (24) 129.5 (120)	<−5.8 (0–<1) −0.5 (0–24) −0.4 (24–120)	DTH (6)	+ (CT, ND)	Diffuse low-density area	Hyperglycemia (max, 371 mg/dL) and hypokalemia (min, 3.4 mEq/L)
17	Miura 2019^[10]^	65 (F)	173	D5W (500; 0–2); HS (60; 2–24) [GL]	Serum	153 (2) 147 (24)	−10 (0–2) −0.3 (2–24)	CR (6)	− (CT, 1) − (MRI, 2)	Normal; Normal	
18	Sakamoto 2020^[26]^	40 (F)	183	NS (1600; 0–1); D5W (600–800; 1–5)	Serum	174 (1) 167 (5) ND	−9.0 (0–1) −1.8 (1–5) −1 to −0.5 (5–)	DTH (8)	− (CT, 1); − (CT, 3)	Brain shrinkage; Brain edema and low-density areas	

CI = cognitive impairment, CR = complete recovery, CT = computed tomography, D2.5W = 2.5% dextrose in water, D5W = 5% dextrose in water, Dex = dexamethasone, DTH = death, FM = furosemide, GL = gastric lavage, HD = hemodialysis, HES = hydroxyethyl starch, HoTS = hypotonic saline, ICH = intracranial hemorrhage, LRS = lactate Ringer solution, MRI = magnetic resonance imaging, Mtl = mannitol, ND = no data, NE = not estimable, NG = nasogastric, NS = normal saline.

∗ Hypernatremia exacerbated due to secondary diabetes insipidus on day 1.

† Administered before the patient's first [Na] became available.

### 3.4 Quality of reporting

The included case reports described well in general about clinical findings, timeline of important events before presentation, and the diagnostic assessment of hypernatremia and its cause (Figure S1, Supplemental Digital Content). However, the quality of reporting was heterogeneous regarding patients’ past medical history and details about treatments, the administration rates of the intravenous fluids in particular. Two case reports lacked sufficient details of patient characteristics^[21]^ and clinical consequences.^[13]^

### 3.5 Mortality and correction rates of hypernatremia

Overall, 5 patients died during a median follow-up of 6 days (range, 2–37 days) (Table 2). Eleven other patients were successfully treated and remained alive during a median follow-up of 7 days (range, 2–90 days). Follow-up days are not reported in the 2 cases (case 1 and 10). All survivors developed no neurological sequelae, except for a single patient treated with hemodialysis who developed cognitive impairment (case 10).

**Figure 2:**
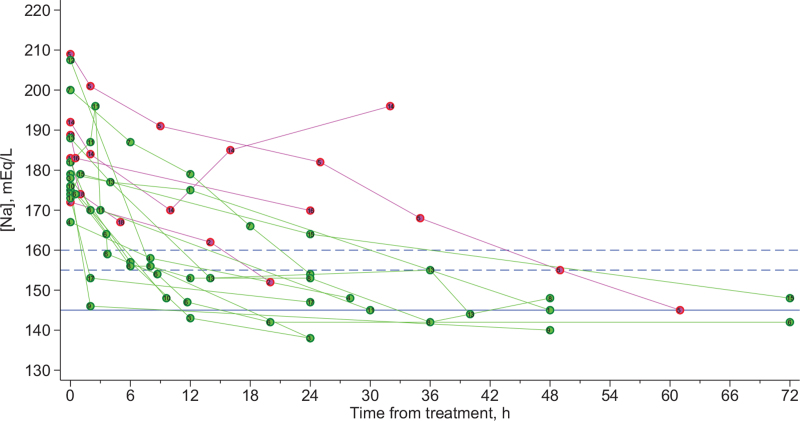
Time-series plot of sodium concentrations ([Na]) in patients with rapid-onset acute hypernatremia. Each circle (green for patients who were successfully treated; magenta for fatal patients) represents sodium concentration measure at a specific timing after the start of treatment. The blue solid horizontal line represents [Na] 145 mEq/L; dashed horizontal lines represent 155 and 160 mEq/L. ∗For individual case id, see Tables 1 and 2 for details.

The time-series plot demonstrated that except for 2 patients who experienced a transient exacerbation (cases 11 and 15), the [Na] in all patients monotonically decreased during the first 6 h (Fig. 2). In 1 patient, who developed diabetes insipidus-induced polyuria due to brain herniation associated with brain edema, a persistent exacerbation of hypernatremia was observed after the first 12 hours (case 14). The mean maximal correction rates were 7.6 mEq/L/h (standard deviation, 13.4) in the 13 successfully treated patients and 4.9 mEq/L/h (standard deviation, 2.7) in the 5 fatal patients. Although the [Na] in the successfully treated group appeared to decrease more steeply than that in the fatal group, the small sample size with the observed clinical heterogeneity, including a wide-ranging [Na]_0_, various fluid regimens, inconsistent timing and frequency of follow-up [Na]s, and various follow-up durations, precluded a standardized, quantitative assessment of the association between the correction rates and mortality.

**Figure 3:**
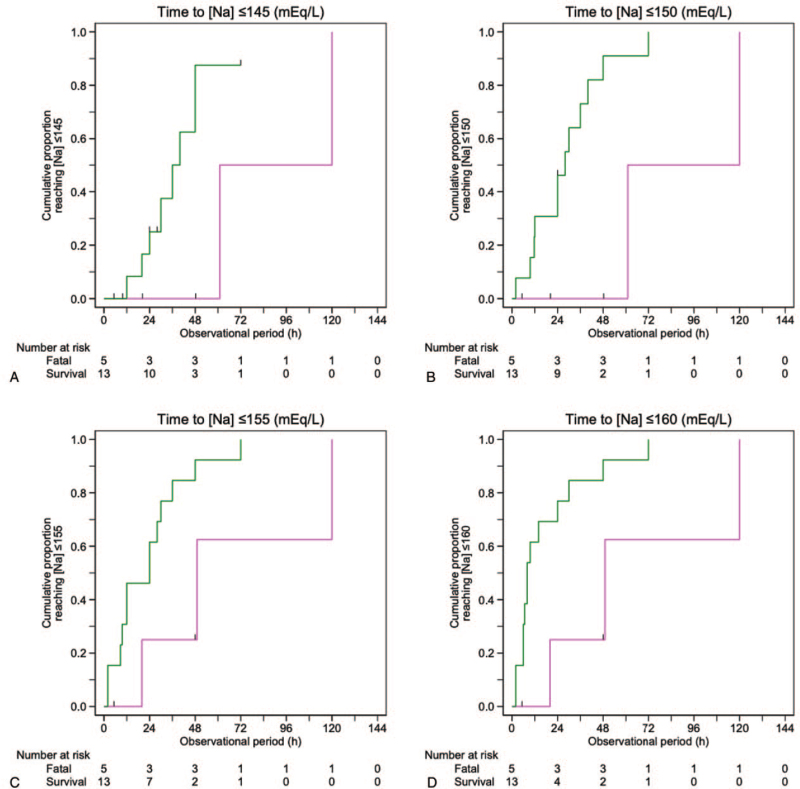
Cumulative proportion of patients who achieved target sodium concentrations ([Na]). The target [Na] for each panel is ≤145 mEq/L (A), ≤150 mEq/L (B), ≤155 mEq/L (C), and ≤160 mEq/L (D). Patients who were successfully treated are plotted in green; patients with fatal outcome are plotted in magenta.

The Kaplan–Meier plot showed that the successfully treated group had a shorter median time to reach [Na] ≤160 than the fatal group (8 hours [interquartile range, IQR: 6–24] in the successfully treated group vs 48 hours [IQR, 20–49] in the fatal group), with 75% in the successfully treated group and 38% in the fatal group achieving [Na] ≤160 at 24 hours (Fig. 3). Similarly, the median time to reach [Na] ≤155 and [Na] ≤150 was shorter in the successfully treated group (24 hours [IQR: 9.6–30] and 24 hours [IQR: 12–36], respectively) than in the fatal group (48 hours [IQR: 20–49] and 48 hours [IQR: 20–61], respectively). Finally, 50% and 90% of the successfully treated patients achieved [Na] ≤145 at 28 and 48 hours, respectively, whereas no patients in the fatal group achieved [Na] ≤145 at 48 hours.

### 3.6 Intracranial hemorrhage and treatment-induced adverse events

A total of 14 patients received postmortem (2 cases) or imaging (computed tomography and/or magnetic resonance imaging, 12 cases) examination of intracranial hemorrhage (Table 2). Intracranial hemorrhage was found in 3 of 5 patients (60%) in the fatal group and none of 8 successfully treated patients (0%). Cerebral edema coexisted in all 3 cases of intracranial hemorrhage; 2 other patients without intracranial hemorrhage also developed cerebral edema.

In general, the reporting of adverse events induced by specific treatments was limited. During the intravenous infusion with dextrose solutions, 7 patients developed hyperglycemia. The maximal glucose levels were 173 to 1116 mg/dL, which required insulin therapy (Table 2). Four patients had mild hypokalemia (2.5–3.4 mEq/L). Although 1 patient reportedly developed postfluid therapy weight gain of 3 kg, no case was documented to develop congestive heart failure secondary to volume overload.

Brain magnetic resonance imaging assessment was only selectively performed; it was performed exclusively in 6 successfully treated cases reported after 2000 (Table 2). Central pontine myelinolysis was found in 2 patients (1 with neurocognitive impairment). All cases were deemed to be induced by hypernatremia per se, rather than by the received treatments.

### 3.7 Sensitivity analysis

**Figure 4:**
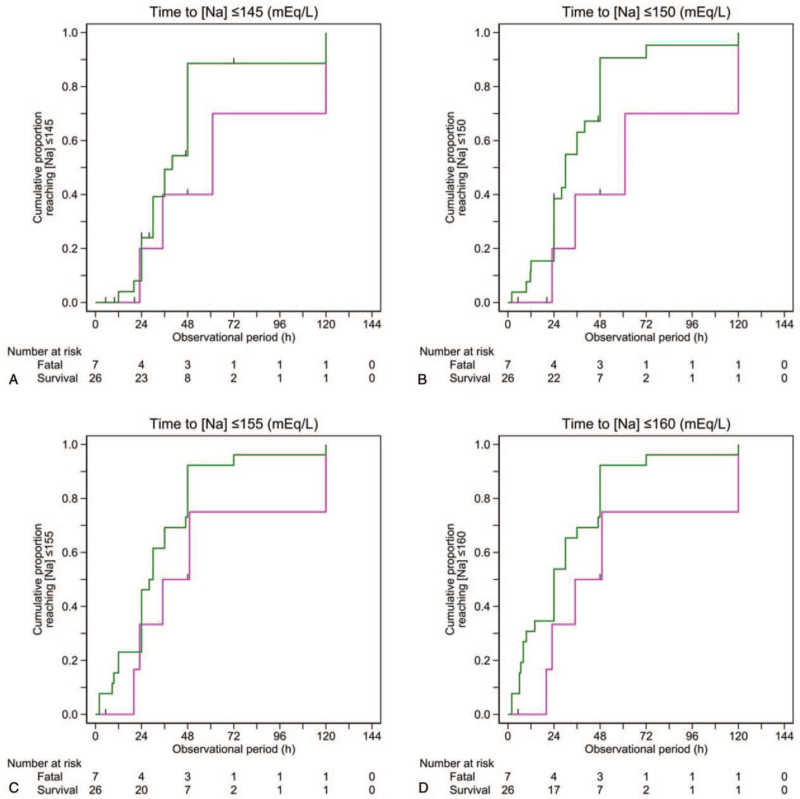
Cumulative proportion of patients who achieved target sodium concentrations ([Na]) (sensitivity analysis). The target [Na] for each panel is ≤145 mEq/L (A), ≤150 mEq/L (B), ≤155 mEq/L (C), and ≤160 mEq/L (D). Patients who were successfully treated are plotted in green; patients with fatal outcome are plotted in magenta. Thirteen case reports with imputed data describing 15 patients are additionally included (see text for details).

Except for 1 patient, the follow-up [Na] data of 15 patients excluded from the primary analysis (due to not explicitly describing both the initial and follow-up (<24 hours) [Na] values) were available only at a single time point (median 35 hours posttreatment, range: 23–48 hours). The sensitivity analysis including these patients resulted in similar cumulative proportion curves for both successfully treated patients and patients who died, irrespective of the target [Na]s set to achieve (Fig. 4).

## 4 Discussion

### 4.1 Primary findings

To our knowledge, this is the first systematic review that has empirically documented the treatments and their clinical outcomes in patients with hypernatremia caused by acute hypertonic sodium gain that developed at <12 hours, typically few hours, after the onset of the cause, based on well-reported 18 case reports. Rapid infusion of dextrose-based solutions, typically D5W, with various initial infusion speeds followed by individually adjusted infusion rates based on follow-up [Na]s, was the most commonly adopted fluid therapy. Hemodialysis was also used where available. The correction rates appeared more rapid in the successfully treated patients than in the fatal patients, although limited data precluded formal quantitative comparisons. The successfully treated patients typically achieved [Na] ≤160 at 8 hours, [Na] ≤150 at 24 hours, and [Na] ≤145 at 28–48 hours. However, these findings were not robust in the sensitivity analysis, where case reports with insufficient data were additionally included. Hyperglycemia was a common treatment-related adverse event in patients treated with dextrose-based regimens.

Brain edema can develop in patients with excessively rapid correction of chronic hypernatremia^[5]^ as it develops in any form of severe brain damage, including intracranial hemorrhage. Besides 3 patients with intracranial hemorrhage and accompanying brain edema, our review found 2 patients who developed brain edema without intracranial hemorrhage. The cause of 1 case was “postictal brain edema”,^[9]^ whereas the authors of the other case discussed a possibility of overcorrection of hypernatremia, similar to a fatal treatment-induced adverse event observed in too rapid correction of chronic hypernatremia.^[26]^ Although both case reports failed to provide concrete evidence to support their interpretations, and thus the etiology remains uncertain, these observations are worth noting, indicating that brain edema can develop in this population without an explicitly identifiable lesion.

### 4.2 Clinical implications

Based on pathophysiological theories, expert recommendations propose rapid correction of acute hypernatremia (defined as hypernatremia that has been present at ≤48 hours) using dextrose-based hypotonic solutions (if available, 2.5% dextrose in water preferred over D5W), with [Na] and blood glucose levels being frequently monitored to adjust the correction speed to target [Na] to decrease to 145 mEq/L within 24 hours.^[6]^ The findings of our systematic review may support this recommendation and its safety as long as the etiology of hypernatremia is hypertonic sodium gain and treatment can be initiated at ≤12 hours from the onset of the cause, although the empirically observed correction rates were slightly slower. Although only 3 patients (reported in 3 case reports) were included, this systematic review may also support the use of hemodialysis for treating hyperacute hypernatremia.^[6]^ Whether the same approach safely applies to similar but different clinical contexts, that is, acute hypernatremia caused by sodium overload where treatment was not started at ≤12 hours or acute hypernatremia secondary to other causes, including unreplaced water losses in general, was not evaluated in this review, and thus remains less clear.

### 4.3 Limitations

Our systematic review has several limitations. As there is no established threshold to distinguish between hyperacute and nonhyperacute forms of acute hypernatremia, our 12-hour cutoff was defined posthoc based on information from reviewed case reports. Moreover, our inferences are only qualitative based on tables and graphs, because we relied on information from a limited number of case reports. Given the small-sized limited data, no quantitative associations could be drawn between specific baseline characteristics and/or treatment methods (and correction rates thereof) and clinical outcomes in these patients. Furthermore, our included case reports may not represent the data in real-life clinical practice; patients with unfavorable outcomes (and also those treated with a particular regimen) may have been systematically left unpublished. However, provided the rarity of this life-threatening condition with an expectedly high mortality, conducting large-scale prospective studies on patients with this condition would be unrealistic. Therefore, despite limited quality, case reports represent a realistic option to empirically document existing evidence. Creation and exploration of a prospective, international consortium registry of this patient group could be another realistic option to further elucidate prognostic factors to predict poor responders to particular regimens, including the recommended treatments, and help develop risk-adapted sodium correction strategies.

## 5 Conclusions

Our limited data add to the existing body of evidence that endorses the current expert-recommended correction approach, that is, rapid and aggressive intravenous administration of 5% dextrose solution to correct [Na] to target 145 mEq/L within 24 hours in patients with hyperacute hypernatremia presenting within 12 hours of sodium overload.

## Author contributions

**Conceptualization:** Takahiro Goshima, Teruhiko Terasawa, Mitsunaga Iwata, Asako Matsushima, Tomonori Hattori, Hiroshi Sasano.

**Data curation:** Takahiro Goshima.

**Formal analysis:** Teruhiko Terasawa.

**Investigation:** Takahiro Goshima.

**Supervision:** Takahiro Goshima, Teruhiko Terasawa, Mitsunaga Iwata, Asako Matsushima, Tomonori Hattori, Hiroshi Sasano.

**Validation:** Takahiro Goshima, Teruhiko Terasawa, Mitsunaga Iwata, Asako Matsushima, Tomonori Hattori, Hiroshi Sasano.

**Writing – original draft:** Takahiro Goshima.

**Writing – review & editing:** Takahiro Goshima, Teruhiko Terasawa, Mitsunaga Iwata, Asako Matsushima, Tomonori Hattori, Hiroshi Sasano.

## Supplementary Material

Supplemental Digital Content

## Supplementary Material

Supplemental Digital Content

## Supplementary Material

Supplemental Digital Content
