# Supplementary material for: Treatment of acute hypernatremia caused by sodium overload in adults: A systematic review
Source: Medicine (Baltimore). 2022 Feb 25;101(8):e28945. doi: 10.1097/MD.0000000000028945 (PMC8878611; doi:10.1097/MD.0000000000028945)
Supplement: Supplemental Digital Content [file medi-101-e28945-s001.docx]

**Supplementary Text**

**Search Strategies**

***PubMed***

hypernatremia

***Ichushi Website***

((高ナトリウム血症/TH or hypernatremia/AL)) and ((PT=症例報告,事例) and (PT=原著論文)
