# Supplementary material for: Treatment of acute hypernatremia caused by sodium overload in adults: A systematic review
Source: Medicine (Baltimore). 2022 Feb 25;101(8):e28945. doi: 10.1097/MD.0000000000028945 (PMC8878611; doi:10.1097/MD.0000000000028945)
Supplement: Supplemental Digital Content [file medi-101-e28945-s002.docx]

### **Supplementary Table 1**. Details of Ineligible Case Series or Case Reports of Patients with Documented Hyperacute Hypernatremia.

| **Author year (Ref.)** | **Age, *years* (sex)** | **Cause of hypernatremia** | **Pre Tx [Na], *mEq/L* (time from ingestion to treatment, *h)*** | **Follow-up [Na], *mEq/L* (post-Tx time, *h*)** | **Clinical outcomes** | **CT/ MRI/ autopsy (post-Tx time, *h*)** | **Findings** | | | **Reasons for exclusion** |  |
| --- | --- | --- | --- | --- | --- | --- | --- | --- | --- | --- | --- |
|  |  |  |  |  |  |  | **Brain edema** | **Brain shrinkage or CPM** | **ICH** |  |  |
| **Studies included in the sensitivity analysis only; excluded from the main analysis because** **the initial and/or follow-up (<24 h) [Na] values were not explicitly reported.** | | | | | | | | | | | |
| Webb 1979-case 4^1^ | 29 (F) | “Salt” | >168 (1) | 140 (<24) correction rates >1 mmol/L/h | Fatal | Autopsy | − | − | + | No explicit initial [Na] value |  |
| Wanninayake 1982^2^ | 44 (M) | Irrigation with NaCl solution in the hydatid operation | 176 (5.25) | “Normal” (24) | Survival | ND | ND | ND | ND | No explicit follow-up [Na] values |  |
| Gage 1984^3^ | 46 (F) | Irrigation with NaCl solution in the hydatid operation | 175 (12) | “Normal” (48) | Survival | CT (ND) | − | − | − | No explicit follow-up [Na] values |  |
| Fujiwara 1985^4^ | 75 (F) | “Chicken soup” | 162 (12) | “Normal” (30) | Survival | ND | ND | ND | ND | No explicit follow-up [Na] values |  |
| Ward 1988^5^ | 66 (F) | Sodium hypochloride | 169 (2) | 142 (24) | Survival | ND | ND | ND | ND | Follow-up [Na] was unavailable <24 h |  |
| Mofredj 2000^6^ | 39 (F) | “Salt solution” | 190 (6) | “Normal” (48) | Survival | ND | ND | ND | ND | No explicit follow-up [Na] values |  |
| Papadimitriou 2001^7^ | 56 (M) | Irrigation with NaCl solution in the hydatid operation | 170 (6) | “Normal” (36) | Survival | ND | ND | ND | ND | No explicit follow-up [Na] values |  |
| Turk 2005-case 1^8^ | 69 (M) | “Salt” | 175 (12) | 140 (<36) | Fatal | Autopsy | + | − | + | Follow-up [Na] was unavailable <36 h |  |
| Machino 2006^9^ | 73 (F) | Soy sauce ingestion | 188 (12) | “Normal” (48) | Survival | MRI (<1 and 3 weeks) | − | + | − | No explicit follow-up [Na] values |  |
| Szolics 2011^10^ | 30 (M) | Irrigation with NaCl solution in the hydatid operation | 183 (10) | 168 (24); “normal” (120) | Survival | CT (48); MRI (72) | + | − | − | Follow-up [Na] was unavailable <24 h |  |
| Wisniewski 2011-case 1^11^ | 50 (F) | “Salt” | 177 (<1) | 135 (24) | Survival | ND | ND | ND | ND | Follow-up [Na] was unavailable <24 h |  |
| Wisniewski 2011-case 2^11^ | 44 (M) | “Salt” | 175 (<1) | 146 (24) | Survival | ND | ND | ND | ND | Follow-up [Na] was unavailable <24 h |  |
| Ju 2013^12^ | 20 (F) | “Salt” | 174 (2~24) | 152 (<48) | Survival | ND | ND | ND | ND | Follow-up [Na] was unavailable <48 h |  |
| Kuzmanovska 2019-case 1^13^ | 17 (F) | Irrigation with NaCl solution in the hydatid operation | 166 (2) | “Normal” (30) | Survival | CT (<48) | − | − | − | No explicit follow-up [Na] values |  |
| Kuzmanovska 2019-case 2^13^ | 70 (M) | Irrigation with NaCl solution in the hydatid operation | 170 (several hours) | Normal (48) | Survival | CT (ND) | − | − | − | No explicit follow-up [Na] values |  |
| **Studies completely excluded because of insufficient data or other reasons.** | | | | | | | | | | |  |
| Cameron 1966^14^ | 20 (F) | NaCl for abortion | 174 (12) | ND | Fatal | autopsy | + | − | − | Follow-up [Na] was not examined. |  |
| Kerenyi 1969^15^ | 19 (F) | NaCl for abortion | 181 (8) | ND6 | Fatal | autopsy | + | − | + | Follow-up [Na] was not examined. |  |
| Robertson 1971^16^ | 23 (F) | “Salt” | 214 (4) | 178 (ND) | Fatal | ND | ND | ND | ND | Insufficient follow-up data. |  |
| Goodbody 1975-case 1^17^ | 44 (F) | “Salt” | 151 (6) | “Normal” (48) | Fatal | Autopsy | + | − | + | Insufficient follow-up data. |  |
| Goodbody 1975-case 2^17^ | 35 (F) | “Salt” | 210 (4) | 226 (postmortem) | Fatal | Autopsy | + | − | − | Insufficient follow-up data. |  |
| Johnston 1977^18^ | 45 (F) | “Salt” | 190 (2–3) | ND | Fatal | Autopsy | − | − | + | Insufficient follow-up data. |  |
| Webb 1979-case 2^1^ | 54 (M) | A large amount of sodium bicarbonate infusion following two cardiac arrests | 168 (ND) | 156 (12) | Fatal | ND | ND | ND | ND | Resuscitated cardiac arrest case |  |
| Webb 1979-case 3^1^ | 69 (M) | “Bladder irrigation with 3.8% sodium citrate” | 162 (9.5) | 160 (5); 155 (14) | Fatal | ND | ND | ND | ND | Insufficient follow-up data. |  |
| Hey 1982^19^ | 56 (F) | “Salt” | 214 (4) | ND | Fatal | ND | ND | ND | ND | Insufficient follow-up data. |  |
| Ofran 2004^20^ | 20 (F) | “Salt” | 255 (11) | ND | Fatal | CT (<24, postmortem) | − | − | − | Insufficient follow-up data. |  |
| Turk 2005-case 2^8^ | 34 (F) | “Salt” | 196 (4.5) | ND | Fatal | Autopsy | + | − | − | Insufficient follow-up data. |  |
| Cobanoglu 2008^21^ | 48 (M) | Irrigation with NaCl solution in the hydatid operation | 185 (3) | ND | Survival | ND | ND | ND | ND | Insufficient follow-up data. |  |
| Buschmann 2010^22^ | 63 (F) | “Salt solution” | 180 (>3–4) | ND | Fatal | ND | ND | ND | ND | Insufficient follow-up data. |  |

# **Supplementary References**

1. Webb A K, Phillips M J and Hanson G C. latrogenic nondiabetic hyperosmolar states. Journal of the Royal Society of Medicine. 1979;72.

2. Wanninayake H M, Brough W, Bullock N et al. Hypernatraemia after treatment of hydatid. Br Med J (Clin Res Ed). 1982 May 1;284(6325):1302-3.

3. Gage T P, Vivian G. Hypernatremia after hypertonic saline irrigation of an hepatic hydatid cyst. Ann Intern Med.1984 Sep 101(3)405.

4. Fujiwara P, Berry M, Hauger P et al. Chicken-soup hypernatremia. N Engl J Med. 1985 Oct 31;313(18):1161-2.

5. Ward M J, Routledge P A. Hypernatraemia and Hyperchloraemic Acidosis After Bleach Ingestion. Human Toxicology. 1988;7:37-38.

6. Mofredj A, Rakotondreantoanina J R, Farouj N. Severe hypernatremia secondary to gastric lavage. Ann Fr Anesth Reanim. 2000 Mar;19(3):219-20. French

7. Papadimitriou L J, Vassiliou J, Katsiamis G et al. An unusual case of iatrogenic severe hypernatremia. Int Surg. Jan-Mar 2001;86(1):49.

8. Türk E E, Schulz F, Koops E et al. Fatal hypernatremia after using salt as an emetic--report of three autopsy cases. Leg Med (Tokyo). 2005 Jan;7(1):47-50.

9. Machino T, Yoshizawa T. Brain shrinkage due to acute hypernatremia. Neurology. 2006 Sep 12;67(5):880.

10. Szólics M, Ljubisavljevic M, Samir H et al. Extrapontine myelinolysis and cortical laminar necrosis caused by severe hypernatremia following peritoneal lavage for ruptured hydatid cyst of the liver. A case report and review of the literature. Neuroradiol J. 2011 May 15;24(2):242-8.

11. Wiśniewski M, Waldman W, Anand J S. Iatrogenic hypernatremia--report of two cases. Przegl Lek. 2011;68(8):557-9. Polish

12. Ju H J, Bae H J, Choi D E et al. Severe hypernatremia by excessive bamboo salt ingestion in healthy young woman. Electrolyte Blood Press. 2013 Dec;11(2):53-5.

13. Kuzmanovska B, Kartalov A, Kuzmanovski I et al. Hypernatremia-induced Neurologic Complications After Hepatic Hydatid Cyst Surgery: Pretreat to Prevent. Med Arch. 2019 Oct;73(5):356-358.

14. Cameron J M, Dayan A D. Association of brain damage with therapeutic abortion induced by amniotic-fluid replacement: report of two cases. Br Med J. 1966 Apr 23;1(5494):1010-3.

15. Kerenyi T D. Hypernatremia following intrauterine instillation of hypertonic saline solution. Report of a case and discussion. Obstet Gynecol. 1969 Apr;33(4):520-7.

16. Robertson W O. A further warning on the use of salt as an emetic agent. J Pediatr. 1971 Nov;79(5):877.

17. Goodbody R A, Middleton J E, Gamlen T R. Saline Emetics and Hypernatraemia: Report on 2 Fatalities. Med Sci Law. 1975 Oct;15(4):261-4.

18. Johnston J G, Robertson W O. Fatal Ingestion of Table Salt by an Adult. West J Med. 1977 Feb;126(2):141-3.

19. Hey A, Hickling K G. Accidental salt poisoning. N Z Med J. 1982 Dec 8;95(721):864.

20. Ofran Y, Lavi D, Opher D et al. Fatal voluntary salt intake resulting in the highest ever documented sodium plasma level in adults (255 mmol L^-1^): a disorder linked to female gender and psychiatric disorders. J Intern Med. 2004 Dec;256(6):525-8.

21. Cobanoğlu U. Postoperative hypernatremia in liver hydatid disease: a case report. Turkiye Parazitol Derg. 2008;32(2):167-70. Turkish

22. Buschmann C T, Lange F, Tsokos M. Fatal sodium chloride intoxication--case report and review of the literature. Arch Kriminol. Jul-Aug 2010;226(1-2):48-54. German

23. Riley D S, Barber M S, Kienle G S et al. CARE guidelines for case reports: explanation and elaboration document. J Clin Epidemiol. 2017 Sep;89:218-235.

24. Heckman B A, Walsh J H. Hypernatremia complicating sodium sulfate therapy for hypercalcemic crisis. N Engl J Med. 1967 May 11;276(19):1082-3.

25. Roberts C J, Noakes M J. Fatal outcome from administration of a salt emetic. Postgrad Med J. 1974 Aug;50(586):513-5.

26. Elisaf M, Litou H, Siamopoulos K C. Survival After Severe latrogenic Hypernatremia. Am J Kidney Dis. 1989 Sep;14(3):230-1.

27. Radonov D, Mirchev N, Madzharov N. Sodium chloride poisoning during abortion. Akush Ginekol (Sofiia). 1989;28(5):77-9. Bulgarian

28. Moder K G, Hurley D L. Fatal Hypernatremia From Exogenous Salt Intake: Report of a Case and Review of the Literature. Mayo Clin Proc. 1990 Dec;65(12):1587-94.

29. Ellis R J. Severe hypernatremia from sea water ingestion during near-drowning in a hurricane. West J Med. 1997 Dec;167(6):430-3.

30. Albi A, Baudin F, Matmar M et al. Severe hypernatremia after hypertonic saline irrigation of hydatid cysts. Anesth Analg. 2002 Dec;95(6):1806-8, table of contents.

31. Ozcan P E, Yavru A, Tuğrul S et al. Iatrogenic hypernatremia during hydatid cyst operation. Ulus Travma Acil Cerrahi Derg. 2003 Oct;9(4):291-3. Turkish

32. Sakai Y, Kato M, Okada T et al. Treatment of salt poisoning due to soy sauce ingestion with hemodialysis. Chudoku Kenkyu. 2004 Jan;17(1):61-3. Japanese

33. Odier C, Nguyen D K, Panisset M. Central pontine and extrapontine myelinolysis: from epileptic and other manifestations to cognitive prognosis. J Neurol. 2010 Jul;257(7):1176-80.

34. Carlberg D J, Borek H A, Syverud S A et al. Survival of acute hypernatremia due to massive soy sauce ingestion. J Emerg Med. 2013 Aug;45(2):228-31.

35. Bhosale G P, Shah V R. Successful recovery from iatrogenic severe hypernatremia and severe metabolic acidosis resulting from accidental use of inappropriate bicarbonate concentrate for hemodialysis treatment. Saudi J Kidney Dis Transpl. 2015 Jan;26(1):107-10.

36. Conde M P S, Rodríguez M Á P, López J M R et al. Thrombosis secondary to acute hypernatraemia after liver hydatid cyst surgery. Blood Coagul Fibrinolysis. 2015 Sep;26(6):695-8.

37. Izutani Y, Morimoto S, Kanayama H et al. A case of intentional massive table salt ingestion. Nihon Kyukyu Igakukai Zasshi: Journal of Japanese Association for Acute Medicine. 2016;27(8):251-5. Japanese

38. Anta D, Beleña J M, Álvarez R et al. Effects of pneumoperitoneum on severe hypernatremia in an adult patient who underwent laparoscopic surgery of hydatid cysts. J Clin Anesth. 2017 Feb;37:52-54.

39. Zeng R, Wu R, Lv Q et al. The association of hypernatremia and hypertonic saline irrigation in hepatic hydatid cysts: A case report and retrospective study. Medicine (Baltimore). 2017 Sep;96(37):e7889.

40. Miura T, Kato H, Inoue S et al. An example of the acute hypernatremia by the soy sauce higher intake. Chuubu journal of acute medicine. 2019;15:35-7. Japanese

41. Sakamoto A, Hoshino T, Boku K et al. Fatal acute hypernatremia resulting from a massive intake of seasoning soy sauce. Acute Med Surg. 2020 Aug 20;7(1):e555.
