# Supplementary material for: Treatment of acute hypernatremia caused by sodium overload in adults: A systematic review
Source: Medicine (Baltimore). 2022 Feb 25;101(8):e28945. doi: 10.1097/MD.0000000000028945 (PMC8878611; doi:10.1097/MD.0000000000028945)
Supplement: Supplemental Digital Content [file medi-101-e28945-s003.docx]

# **Supplementary Figure 1**. Quality of Reporting Based on the CARE Guidelines^23^

|  | **Patient Information** | a) Demographic information (e.g., age, gender, ethnicity, occupation) | b) Main symptoms of the patient (his or her chief complaints) | c) Medical, family, and psychosocial history | **Clinical Findings** | Description of the relevant physical examination (PE) findings | **Timeline** | Depiction of important dates and times in this case (table or figure) | **Diagnostic Assessment** | a) Diagnostic methods (e.g., PE, laboratory testing, imaging, questionnaires) | b) Diagnostic challenges (e.g., financial, language/cultural) | c) Diagnostic reasoning including other diagnoses considered | d) Prognostic characteristics (e.g., staging) where applicable | **Therapeutic Intervention** | Types of intervention (e.g., pharmacologic, surgical, preventive, self-care) | Administration of intervention (e.g., dosage, strength, duration) | Changes in intervention (with rationale) | **Follow-up and Outcomes** | Clinician- and patient-assessed outcomes | Important follow-up test results (positive or negative) | Intervention adherence and tolerability (and how this was assessed) | Adverse and unanticipated events | **Overall** |
| --- | --- | --- | --- | --- | --- | --- | --- | --- | --- | --- | --- | --- | --- | --- | --- | --- | --- | --- | --- | --- | --- | --- | --- |
| **Included studies** | | | | | | | | | | | | | | | | | | | | | | | |
| Heckman 1967^24^ | 😐 | Y | Y | PY | ☺ | Y | 😐 | Y | ☺ | Y | NA | NA | NA | 😐 | Y | PY | Y | ☹ | Y | N | NA | Y | 😐 |
| Roberts 1974^25^ | ☺ | Y | Y | Y | ☺ | Y | ☺ | Y | ☺ | Y | NA | NA | NA | ☺ | Y | Y | Y | 😐 | Y | NA | NA | U | ☺ |
| Elisaf 1989^26^ | 😐 | Y | Y | PY | ☺ | Y | ☺ | Y | ☺ | Y | NA | NA | NA | ☺ | Y | Y | Y | ☺ | Y | Y | NA | Y | ☺ |
| Radonov 1989^27^ | 😐 | Y | Y | PY | ☺ | Y | ☺ | Y | ☺ | Y | NA | NA | NA | 😐 | Y | PY | Y | 😐 | Y | Y | NA | U | ☺ |
| Moder 1990^28^ | ☺ | Y | Y | Y | ☺ | Y | ☺ | Y | ☺ | Y | NA | NA | NA | ☺ | Y | Y | Y | ☺ | Y | NA | NA | U | ☺ |
| Ellis 1997^29^ | ☺ | Y | Y | Y | ☺ | Y | ☺ | Y | ☺ | Y | NA | NA | NA | 😐 | Y | PY | Y | 😐 | Y | Y | NA | U | ☺ |
| Albi 2002^30^ | 😐 | Y | Y | U | ☺ | Y | ☺ | Y | ☺ | Y | NA | NA | NA | ☺ | Y | Y | Y | ☺ | Y | Y | NA | Y | ☺ |
| Ozcan 2003^31^ | ☺ | Y | Y | PY | ☺ | Y | ☺ | Y | ☺ | Y | NA | NA | NA | ☺ | Y | Y | Y | 😐 | Y | PY | NA | Y | ☺ |
| Sakai 2004^32^ | 😐 | Y | Y | PY | ☺ | Y | ☺ | Y | ☺ | Y | NA | NA | NA | ☺ | Y | Y | Y | 😐 | Y | Y | NA | U | 😐 |
| Odier 2010^33^ | ☹ | Y | N | N | ☹ | N | ☺ | Y | ☹ | N | NA | NA | NA | 😐 | Y | PY | U | 😐 | Y | Y | NA | U | 😐 |
| Carlberg 2013^34^ | 😐 | Y | Y | PY | ☺ | Y | ☺ | Y | ☺ | Y | NA | NA | NA | ☺ | Y | Y | Y | ☺ | Y | Y | NA | Y | ☺ |
| Bhosale 2015^35^ | 😐 | Y | Y | PY | ☺ | Y | ☺ | Y | ☺ | Y | NA | NA | NA | 😐 | Y | Y | Y | ☺ | Y | Y | NA | Y | ☺ |
| Conde 2015^36^ | 😐 | Y | Y | PY | ☺ | Y | ☺ | Y | ☺ | Y | NA | NA | NA | ☺ | Y | Y | Y | ☺ | Y | Y | NA | Y | ☺ |
| Izutani 2016^37^ | ☺ | Y | Y | Y | ☺ | Y | ☺ | Y | ☺ | Y | NA | NA | NA | ☺ | Y | Y | Y | ☺ | Y | NA | NA | Y | ☺ |
| Anta 2017^38^ | ☺ | Y | Y | Y | 😐 | PY | ☺ | Y | ☺ | Y | NA | NA | NA | 😐 | Y | PY | Y | ☺ | Y | Y | NA | Y | 😐 |
| Zeng 2017^39^ | ☺ | Y | Y | Y | 😐 | PY | ☺ | Y | ☺ | Y | NA | NA | NA | 😐 | Y | PY | Y | ☺ | Y | NA | NA | Y | 😐 |
| Miura 2019^40^ | ☺ | Y | Y | Y | ☺ | Y | ☺ | Y | ☺ | Y | NA | NA | NA | ☺ | Y | Y | Y | 😐 | Y | Y | NA | U | ☺ |
| Sakamoto 2020^41^ | ☺ | Y | Y | Y | ☺ | Y | 😐 | Y | ☺ | Y | NA | NA | NA | 😐 | Y | PY | Y | 😐 | Y | NA | NA | U | 😐 |
| **Studies included in the sensitivity analysis only** | | | | | | | | | | | | | | | | | | | | | | | |
| Webb 1979-case 4 (29F)^1^ | 😐 | Y | Y | PY | ☺ | Y | 😐 | PY  (graph) | ☺ | Y | NA | NA | NA | ☺ | Y | Y | Y | 😐 | Y | NA | NA | U | 😐 |
| Wanninayake 1982^2^ | ☹ | Y | Y | N | ☺ | Y | 😐 | PY  (graph) | 😐 | PY | NA | NA | NA | ☺ | Y | Y | Y | ☹ | Y | N | NA | U | 😐 |
| Gage 1984^3^ | ☹ | Y | Y | N | 😐 | PY | 😐 | PY | 😐 | PY | NA | NA | NA | 😐 | Y | PY | Y | ☹ | Y | N | NA | U | 😐 |
| Fujiwara 1985^4^ | 😐 | Y | Y | PY | ☺ | Y | 😐 | PY | 😐 | PY | NA | NA | NA | 😐 | Y | PY | U | ☹ | Y | N | NA | U | 😐 |
| Ward 1988^5^ | 😐 | Y | Y | PY | 😐 | PY | 😐 | PY | ☺ | Y | NA | NA | NA | ☹ | Y | N | U | 😐 | Y | Y | NA | U | 😐 |
| Mofredj 2000^6^ | 😐 | Y | Y | PY | ☺ | Y | 😐 | PY | ☺ | Y | NA | NA | NA | 😐 | Y | PY | U | 😐 | Y | Y | NA | U | 😐 |
| Papadimitriou 2001^7^ | ☹ | Y | Y | N | ☺ | Y | 😐 | PY | 😐 | PY | NA | NA | NA | ☹ | Y | N | U | 😐 | Y | Y | NA | U | 😐 |
| Turk 2005-case 1^8^ | 😐 | Y | Y | PY | 😐 | PY | 😐 | PY | 😐 | PY | NA | NA | NA | ☹ | N | N | U | 😐 | Y | NA | NA | U | 😐 |
| Machino 2006^9^ | ☹ | Y | Y | N | ☹ | N | 😐 | PY | 😐 | PY | NA | NA | NA | ☹ | N | N | U | 😐 | Y | Y | NA | U | 😐 |
| Szolics 2011^10^ | ☹ | Y | Y | N | ☺ | Y | 😐 | PY  (graph) | ☺ | PY | NA | NA | NA | 😐 | Y | PY | U | 😐 | Y | Y | NA | U | 😐 |
| Wisniewski 2011-1 (50F)^11^ | ☹ | Y | Y | N | ☺ | Y | 😐 | PY | 😐 | Y | NA | NA | NA | 😐 | Y | PY | U | 😐 | Y | Y | NA | U | 😐 |
| Wisniewski 2011-2 (44M)^11^ | ☹ | Y | Y | N | ☺ | Y | 😐 | PY | 😐 | Y | NA | NA | NA | 😐 | Y | PY | U | 😐 | Y | Y | NA | U | 😐 |
| Ju 2013^12^ | 😐 | Y | Y | PY | ☺ | Y | 😐 | PY  (graph) | ☺ | Y | NA | NA | NA | 😐 | Y | Y | U | 😐 | Y | Y | NA | U | 😐 |
| Kuzmanovska 2019-1 (17F)^13^ | 😐 | Y | Y | PY | 😐 | PY | 😐 | PY | 😐 | PY | NA | NA | NA | 😐 | Y | PY | U | 😐 | PY | PY | NA | U | 😐 |
| Kuzmanovska 2019-2 (70M)^13^ | 😐 | Y | Y | PY | 😐 | PY | 😐 | PY | 😐 | PY | NA | NA | NA | 😐 | Y | PY | U | 😐 | Y | Y | NA | U | 😐 |
| **Studies completely excluded** | | | | | | | | | | | | | | | | | | | | | | | |
| Cameron 1966^14^ | 😐 | Y | Y | PY | 😐 | PY | ☹ | N | 😐 | PY | NA | NA | NA | ☹ | PY | N | U | 😐 | Y | NA | NA | U | ☹ |
| Kerenyi 1969^15^ | 😐 | Y | Y | PY | ☺ | Y | ☹ | N | ☺ | Y | NA | NA | NA | 😐 | Y | PY | Y | 😐 | Y | NA | NA | U | ☹ |
| Robertson 1971^16^ | 😐 | Y | U | U | ☹ | N | ☹ | N | 😐 | PY | NA | NA | NA | ☹ | N | N | N | 😐 | Y | NA | NA | U | ☹ |
| Goodbody 1975-case 1^17^ | 😐 | Y | Y | PY | 😐 | PY | ☹ | N | 😐 | PY | NA | NA | NA | ☹ | N | N | N | 😐 | Y | NA | NA | U | ☹ |
| Goodbody 1975-case 2^17^ | 😐 | Y | Y | PY | 😐 | PY | 😐 | PY | 😐 | PY | NA | NA | NA | ☹ | N | N | N | 😐 | Y | NA | NA | U | ☹ |
| Johnston 1977^18^ | ☺ | Y | Y | Y | ☺ | Y | ☹ | N | ☺ | Y | NA | NA | NA | ☹ | PY | N | N | 😐 | Y | NA | NA | U | ☹ |
| Webb 1979-case 2^1^ | 😐 | Y | Y | PY | ☹ | N | 😐 | PY | ☺ | Y | NA | NA | NA | ☹ | N | N | N | 😐 | Y | NA | NA | U | ☹ |
| Webb 1979-case 3^1^ | 😐 | Y | Y | PY | 😐 | PY | 😐 | PY | ☺ | Y | NA | NA | NA | ☹ | Y | N | Y | 😐 | Y | NA | NA | U | ☹ |
| Hey 1982^19^ | ☹ | Y | Y | N | 😐 | PY | ☹ | N | 😐 | PY | NA | NA | NA | ☹ | Y | N | U | 😐 | Y | NA | NA | U | ☹ |
| Ofran 2004^20^ | 😐 | Y | Y | PY | ☺ | Y | ☹ | N | 😐 | PY | NA | NA | NA | ☹ | Y | N | U | 😐 | Y | NA | NA | U | ☹ |
| Turk 2005-case 2^8^ | 😐 | Y | Y | PY | 😐 | PY | ☹ | N | 😐 | PY | NA | NA | NA | ☹ | Y | N | U | 😐 | Y | NA | NA | U | ☹ |
| Cobanoglu 2008^21^ | 😐 | Y | Y | PY | 😐 | PY | ☹ | N | ☺ | Y | NA | NA | NA | ☹ | Y | N | U | 😐 | Y | Y | NA | U | ☹ |
| Buschmann 2010^22^ | 😐 | Y | Y | PY | 😐 | PY | ☹ | N | 😐 | PY | NA | NA | NA | ☹ | N | N | U | 😐 | Y | Y | NA | U | ☹ |

Key

| ☺ | Good |
| --- | --- |
| 😐 | Fair |
| ☹ | Poor |

CARE = Consensus-based Clinical Case Reporting; N = not reported; NA = not applicable; PY = partially reported; U = unclear; Y = reported.

# **Supplementary References**

1. Webb A K, Phillips M J and Hanson G C. latrogenic nondiabetic hyperosmolar states. Journal of the Royal Society of Medicine. 1979;72.

2. Wanninayake H M, Brough W, Bullock N et al. Hypernatraemia after treatment of hydatid. Br Med J (Clin Res Ed). 1982 May 1;284(6325):1302-3.

3. Gage T P, Vivian G. Hypernatremia after hypertonic saline irrigation of an hepatic hydatid cyst. Ann Intern Med.1984 Sep 101(3)405.

4. Fujiwara P, Berry M, Hauger P et al. Chicken-soup hypernatremia. N Engl J Med. 1985 Oct 31;313(18):1161-2.

5. Ward M J, Routledge P A. Hypernatraemia and Hyperchloraemic Acidosis After Bleach Ingestion. Human Toxicology. 1988;7:37-38.

6. Mofredj A, Rakotondreantoanina J R, Farouj N. Severe hypernatremia secondary to gastric lavage. Ann Fr Anesth Reanim. 2000 Mar;19(3):219-20. French

7. Papadimitriou L J, Vassiliou J, Katsiamis G et al. An unusual case of iatrogenic severe hypernatremia. Int Surg. Jan-Mar 2001;86(1):49.

8. Türk E E, Schulz F, Koops E et al. Fatal hypernatremia after using salt as an emetic--report of three autopsy cases. Leg Med (Tokyo). 2005 Jan;7(1):47-50.

9. Machino T, Yoshizawa T. Brain shrinkage due to acute hypernatremia. Neurology. 2006 Sep 12;67(5):880.

10. Szólics M, Ljubisavljevic M, Samir H et al. Extrapontine myelinolysis and cortical laminar necrosis caused by severe hypernatremia following peritoneal lavage for ruptured hydatid cyst of the liver. A case report and review of the literature. Neuroradiol J. 2011 May 15;24(2):242-8.

11. Wiśniewski M, Waldman W, Anand J S. Iatrogenic hypernatremia--report of two cases. Przegl Lek. 2011;68(8):557-9. Polish

12. Ju H J, Bae H J, Choi D E et al. Severe hypernatremia by excessive bamboo salt ingestion in healthy young woman. Electrolyte Blood Press. 2013 Dec;11(2):53-5.

13. Kuzmanovska B, Kartalov A, Kuzmanovski I et al. Hypernatremia-induced Neurologic Complications After Hepatic Hydatid Cyst Surgery: Pretreat to Prevent. Med Arch. 2019 Oct;73(5):356-358.

14. Cameron J M, Dayan A D. Association of brain damage with therapeutic abortion induced by amniotic-fluid replacement: report of two cases. Br Med J. 1966 Apr 23;1(5494):1010-3.

15. Kerenyi T D. Hypernatremia following intrauterine instillation of hypertonic saline solution. Report of a case and discussion. Obstet Gynecol. 1969 Apr;33(4):520-7.

16. Robertson W O. A further warning on the use of salt as an emetic agent. J Pediatr. 1971 Nov;79(5):877.

17. Goodbody R A, Middleton J E, Gamlen T R. Saline Emetics and Hypernatraemia: Report on 2 Fatalities. Med Sci Law. 1975 Oct;15(4):261-4.

18. Johnston J G, Robertson W O. Fatal Ingestion of Table Salt by an Adult. West J Med. 1977 Feb;126(2):141-3.

19. Hey A, Hickling K G. Accidental salt poisoning. N Z Med J. 1982 Dec 8;95(721):864.

20. Ofran Y, Lavi D, Opher D et al. Fatal voluntary salt intake resulting in the highest ever documented sodium plasma level in adults (255 mmol L^-1^): a disorder linked to female gender and psychiatric disorders. J Intern Med. 2004 Dec;256(6):525-8.

21. Cobanoğlu U. Postoperative hypernatremia in liver hydatid disease: a case report. Turkiye Parazitol Derg. 2008;32(2):167-70. Turkish

22. Buschmann C T, Lange F, Tsokos M. Fatal sodium chloride intoxication--case report and review of the literature. Arch Kriminol. Jul-Aug 2010;226(1-2):48-54. German

23. Riley D S, Barber M S, Kienle G S et al. CARE guidelines for case reports: explanation and elaboration document. J Clin Epidemiol. 2017 Sep;89:218-235.

24. Heckman B A, Walsh J H. Hypernatremia complicating sodium sulfate therapy for hypercalcemic crisis. N Engl J Med. 1967 May 11;276(19):1082-3.

25. Roberts C J, Noakes M J. Fatal outcome from administration of a salt emetic. Postgrad Med J. 1974 Aug;50(586):513-5.

26. Elisaf M, Litou H, Siamopoulos K C. Survival After Severe latrogenic Hypernatremia. Am J Kidney Dis. 1989 Sep;14(3):230-1.

27. Radonov D, Mirchev N, Madzharov N. Sodium chloride poisoning during abortion. Akush Ginekol (Sofiia). 1989;28(5):77-9. Bulgarian

28. Moder K G, Hurley D L. Fatal Hypernatremia From Exogenous Salt Intake: Report of a Case and Review of the Literature. Mayo Clin Proc. 1990 Dec;65(12):1587-94.

29. Ellis R J. Severe hypernatremia from sea water ingestion during near-drowning in a hurricane. West J Med. 1997 Dec;167(6):430-3.

30. Albi A, Baudin F, Matmar M et al. Severe hypernatremia after hypertonic saline irrigation of hydatid cysts. Anesth Analg. 2002 Dec;95(6):1806-8, table of contents.

31. Ozcan P E, Yavru A, Tuğrul S et al. Iatrogenic hypernatremia during hydatid cyst operation. Ulus Travma Acil Cerrahi Derg. 2003 Oct;9(4):291-3. Turkish

32. Sakai Y, Kato M, Okada T et al. Treatment of salt poisoning due to soy sauce ingestion with hemodialysis. Chudoku Kenkyu. 2004 Jan;17(1):61-3. Japanese

33. Odier C, Nguyen D K, Panisset M. Central pontine and extrapontine myelinolysis: from epileptic and other manifestations to cognitive prognosis. J Neurol. 2010 Jul;257(7):1176-80.

34. Carlberg D J, Borek H A, Syverud S A et al. Survival of acute hypernatremia due to massive soy sauce ingestion. J Emerg Med. 2013 Aug;45(2):228-31.

35. Bhosale G P, Shah V R. Successful recovery from iatrogenic severe hypernatremia and severe metabolic acidosis resulting from accidental use of inappropriate bicarbonate concentrate for hemodialysis treatment. Saudi J Kidney Dis Transpl. 2015 Jan;26(1):107-10.

36. Conde M P S, Rodríguez M Á P, López J M R et al. Thrombosis secondary to acute hypernatraemia after liver hydatid cyst surgery. Blood Coagul Fibrinolysis. 2015 Sep;26(6):695-8.

37. Izutani Y, Morimoto S, Kanayama H et al. A case of intentional massive table salt ingestion. Nihon Kyukyu Igakukai Zasshi: Journal of Japanese Association for Acute Medicine. 2016;27(8):251-5. Japanese

38. Anta D, Beleña J M, Álvarez R et al. Effects of pneumoperitoneum on severe hypernatremia in an adult patient who underwent laparoscopic surgery of hydatid cysts. J Clin Anesth. 2017 Feb;37:52-54.

39. Zeng R, Wu R, Lv Q et al. The association of hypernatremia and hypertonic saline irrigation in hepatic hydatid cysts: A case report and retrospective study. Medicine (Baltimore). 2017 Sep;96(37):e7889.

40. Miura T, Kato H, Inoue S et al. An example of the acute hypernatremia by the soy sauce higher intake. Chuubu journal of acute medicine. 2019;15:35-7. Japanese

41. Sakamoto A, Hoshino T, Boku K et al. Fatal acute hypernatremia resulting from a massive intake of seasoning soy sauce. Acute Med Surg. 2020 Aug 20;7(1):e555.
